# MIND the translational gap: Preclinical models of ductal carcinoma in situ

**DOI:** 10.1002/ctm2.1376

**Published:** 2023-08-24

**Authors:** Stefan J. Hutten, Jos Jonkers

**Affiliations:** ^1^ Division of Molecular Pathology Oncode Institute, Netherlands Cancer Institute Amsterdam The Netherlands

## 1 INTRODUCTION

Ductal carcinoma in situ (DCIS) is a non‐invasive premalignant lesion and is considered a non‐obligate precursor of invasive breast cancer (IBC). DCIS has increasingly been diagnosed due to screening efforts, but treatment of patients with DCIS has not led to a proportional decrease of late stage disease.^1^ As all DCIS patients are treated, only a limited number of studies were able to follow‐up on untreated DCIS due to misdiagnosis of biopsies, showing that only 30%−60% of all DCIS cases will progress to invasive disease.^2^, ^3^, ^4^, ^5^, ^6^, ^7^ Unfortunately, we are unable to predict which DCIS lesions remain indolent and which lesions progress to invasive disease. Therefore, the current standard of care for women diagnosed with DCIS remains breast‐conserving surgery followed by radiotherapy or mastectomy, with a subset of patients receiving adjuvant endocrine therapy.^8^ As it is suspected that the majority of DCIS lesions will never progress to invasive disease, a large group of women are at risk of overtreatment. It is therefore crucial to better understand the mechanisms driving invasive progression of DCIS.

**FIGURE 1:**
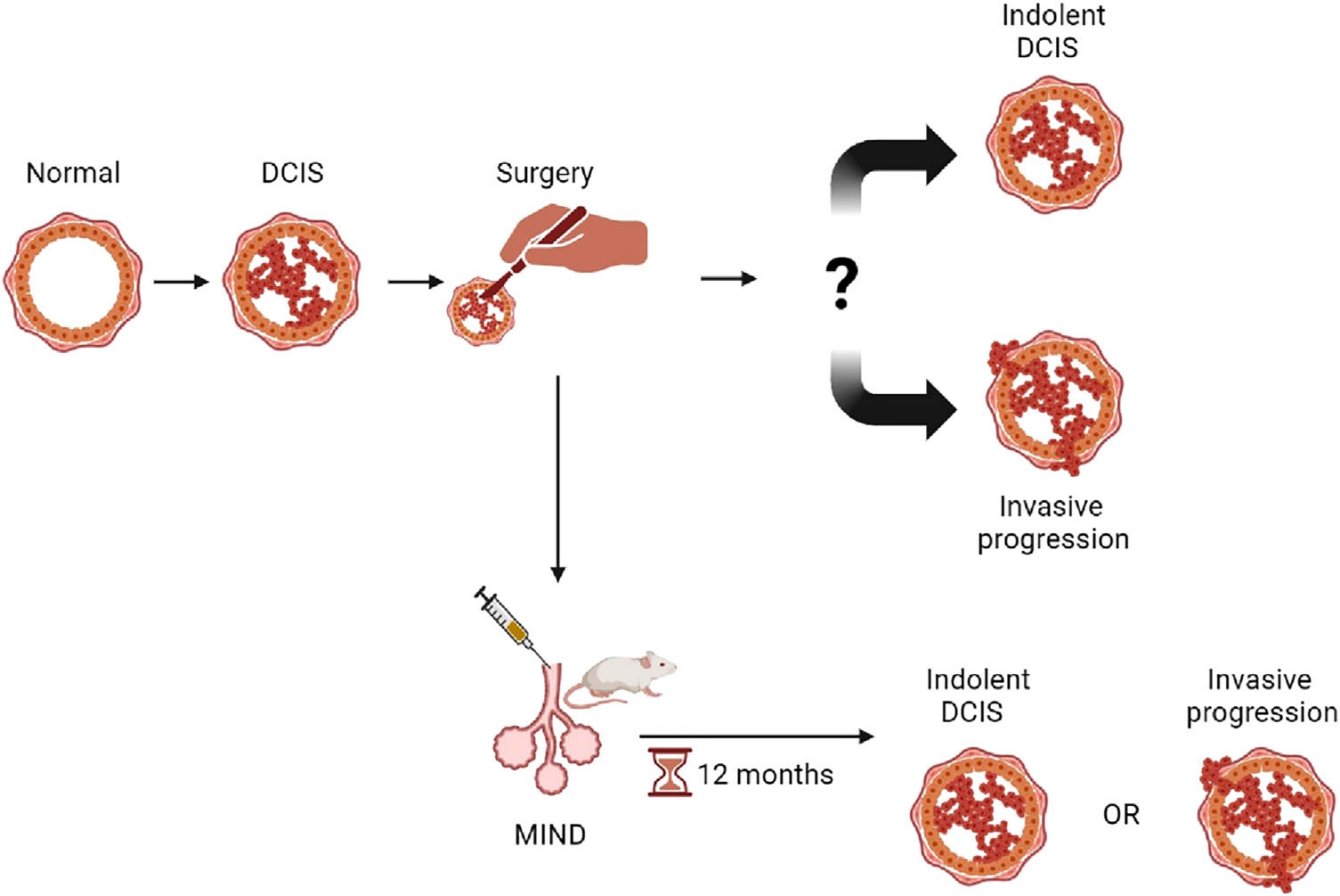
Schematic overview of the DCIS conundrum, showing DCIS‐MIND models as a possible solution to better understand DCIS progression. DCIS, ductal carcinoma in situ; MIND, Mouse INtraDuctal.

It is difficult to study DCIS progression in patients because most women with DCIS receive surgery. Consequently, most human studies have focused on synchronous DCIS‐IBC to identify genetic differences; however, these studies mainly identified high genetic concordance between DCIS and IBC.^9^, ^10^, ^11^ As these studies only allow investigation of DCIS with proven invasive potential and do not allow investigation of the early disease stages, it is questionable how informative they are. Several risk classifiers have been developed for DCIS, such as the Oncotype DX DCIS and 812‐gene classifier.^12^, ^13^ However, these classifiers focus on disease recurrence rather than on risk of DCIS progression without treatment. Therefore, there is a need for preclinical model systems that recapitulate DCIS progression and capture the molecular and genetic heterogeneity of DCIS. The ideal model should be easy to work with, recapitulate both indolent as DCIS with invasive potential, mimic the human microenvironment, and capture the full molecular spectrum of DCIS. It is specifically important that models also represent luminal A and luminal B subtypes as they account for about 50% and 20%, respectively, of all DCIS cases, whereas HER2 amplified accounts for 25% and the rare triple negative DCIS subtype only accounts for 5%, as they rapidly progress to invasive disease.^14^ Several DCIS model systems have been developed, including genetically engineered mouse models (GEMMs), cell line‐derived xenograft (CDX) models, syngeneic mouse models, and Mouse INtraDuctal (MIND) xenograft models based on intraductal injection of patient‐derived primary DCIS cells in immunodeficient mice. Especially the latter has proven to be a useful model system to study DCIS progression (Figure 1). Here, we will describe the different model systems and how well they approximate the ideal preclinical DCIS model to bridge the translational gap.

## 2 GENETICALLY ENGINEERED MOUSE MODELS

Many GEMMs have been developed for breast cancer, but only a few of these models show progression through pre‐invasive lesions resembling DCIS. The most well‐known GEMM that recapitulates DCIS progression is the MMTV‐PyMT transgenic mouse. The mammary glands of these mice show hyperplasia at 4 weeks, DCIS‐like lesions at 8–9 weeks, and invasive disease from 10 weeks onwards.^15^ MMTV‐PyMT mice show partial oestrogen receptor (OR) expression in the early stages, but finally progress to OR‐negative IBC. Other MMTV‐transgenic mice that progress through pre‐invasive lesions are MMTV‐*ErbB2/Neu* and MMTV‐iFGFR1 mice. MMTV‐ErbB2/neu mice represent OR‐negative/HER2‐positive disease and present with solid DCIS lesions that progress to invasive disease after 12–14 weeks, whereas MMTV‐iFGFR1 transgenic mice present with multicellular mammary epithelium with small collapsed lumens 2 weeks after FGFR1 induction and invasive progression at 4 weeks after FGFR1 induction.^16^, ^17^ Finally, the C3(1)/SV40 T‐antigen [C3(1)/TAg] transgenic mouse shows ductal atypia at 8 weeks, lesions representing a basal subtype of DCIS at 12 weeks, and invasive progression at 16 weeks.^18^, ^19^

These GEMMs provide a great opportunity to study the different stages of mammary tumour initiation and progression, but they also have important limitations. First of all, the time span from pre‐invasive to invasive disease is weeks, whereas human DCIS can take months or years to progress to invasive disease. Furthermore, mammary tumours arising in GEMMs mainly represent OR‐negative disease. Herschkowitz et al. characterised 13 commonly used GEMMs, including MMTV‐PyMT, MMTV‐Neu, and C3(1)/TAg and showed that none of the models represent a luminal A subtype, even though 50%−60% of DCIS lesions classify as luminal A.^20^ This suggests that GEMMs mainly recapitulate more aggressive subtypes of breast cancer, with rapid progression to IDC.

## 3 SYNGENEIC MOUSE MODELS

To study neoplastic progression, a transplantable model of human DCIS was generated by deriving transplantable mammary intraepithelial neoplasia outgrowth (MIN‐O) lines from early dysplastic lesions from MMTV‐PyMT mice. Transplantation of these MIN‐O lines into cleared mammary fat pads of immunocompetent syngeneic host female mice results in dysplastic outgrowths after 5–6 weeks and palpable tumours after 11–22 weeks, depending on the MIN‐O line used.^21^ Similar results have been obtained with preneoplastic outgrowth lines derived from p53‐null mouse mammary glands.^22^ Outgrowth and progression of MIN‐O lines is delayed by selective OR modulators, suggesting that the MIN‐O model might be useful for preclinical evaluation of chemoprevention therapies.^23^

While the MIN‐O model enables researchers to study progression of preneoplastic lesions in an immunocompetent setting, this model suffers from the same limitations as the above‐mentioned MMTV‐PyMT GEMM from which it was derived, that is rapid progression to invasive triple‐negative mammary tumours and failure to recapitulate the heterogeneity observed in human DCIS.

## 4 CELL LINE‐DERIVED XENOGRAFT MODELS

Another commonly used platform to study DCIS progression is CDX models based on the MIND injection method developed by Behbod et al.^22^ With this method, human cells are injected directly into the ducts of immunocompromised mice. This ensures the initiation site and micro‐environment mimic the human setting as much as possible. Using this method, Sflomos et al. have shown that OR+ breast cancer cell lines such as MCF7 and T47D grow as DCIS before progressing to invasive disease. Importantly, the microenvironment in the milk ducts ensures the tumour cells retain their luminal subtype by suppressing SLUG expression, whereas TGFβ/SLUG signalling in the fat pad microenvironment causes basal differentiation.^24^ This shows the MIND model has the potential to mimic human DCIS characteristics and progression.

As most breast cancer cell lines such as MCF7 and T47D are derived from metastatic or invasive disease, it is important to obtain models representing less advanced stages of breast cancer. Currently, the most frequently used MIND‐CDX models representing DCIS employ MCF10DCIS.com and SUM225 cells. MCF10DCIS.com was cloned from xenograft lesions from the normal‐like breast epithelial cell line MCF10AT, which has been transfected with the *c‐Ha‐ras* oncogene.^25^ MCF10DCIS.com gives rise to cribriform DCIS lesions, which progress to invasive disease after 6 weeks and are triple negative (i.e. they lack expression of OR, PR, and HER2).^26^, ^27^ The SUM225 cell line originates from a chest wall recurrence of DCIS, which lacks expression of OR and PR, but has HER2 overexpression. SUM225 MIND models grow as solid DCIS lesions with comedonecrosis and become invasive 14 weeks after intraductal injection.^27^, ^28^ While these two cell lines have been used as the gold standard for studying DCIS progression in vivo, it remains questionable how well they represent the majority of DCIS lesions. Triple‐negative and HER2‐amplified DCIS represent only 5% and 25% of all DCIS cases, respectively, and are believed to be the most aggressive subtypes, which is also reflected by the fact that both CDX models show rapid progression to invasive disease.^14^

In sum, CDX models show that MIND modelling can faithfully recapitulate DCIS formation and the ability to retain different molecular subtypes, including a luminal subtype. Unfortunately, the currently available CDX models fail to recapitulate the full spectrum of DCIS in patients, because they do not capture the more indolent class of DCIS lesions.

## 5 PRIMARY XENOGRAFT MODELS

GEMMs, syngeneic transplantation models, and CDX models have been useful in studying the relatively uncommon subtypes of DCIS (TN and OR–/HER2+). However, they fail to effectively bridge the translational gap because they do not recapitulate OR+/HER2– DCIS, which represents the majority of all cases. Hence, there is an unmet need for model systems that capture the full heterogeneity of DCIS lesions and are able to mimic indolent DCIS.

Previous studies have shown that MIND enables modelling of OR+ breast cancer, as well as primary DCIS lesions.^24^, ^29^ Therefore, we set out to create a large biobank of DCIS‐MIND models covering the full heterogeneity of DCIS lesions in patients. In our recent study,^30^ we obtained DCIS tissue from 130 primary surgeries and engrafted them as single cells in immunodeficient *NOD‐scid;Il2rg^null^* (NSG) mice using the MIND method, with a take rate of 88%. By following the outgrowth of these lesions over a 12‐month time period, we created a biobank of 115 DCIS‐MIND models encompassing DCIS lesions with all different growth patterns, molecular subtypes, and grades and effectively recapitulating the patients’ DCIS lesions. Importantly, this collection of models allowed us to follow the natural progression of DCIS lesions, showing that 46% of DCIS lesions progress to invasive disease, indicating that approximately half of all DCIS patients might not require any treatment. By combining the outcome data with clinicopathological features and multi‐omics data, we could identify multiple prognostic factors for high‐risk DCIS, including high grade, HER2 amplification, and a high burden of DNA copy number aberrations. Moreover, we were able to show that recurrence classifiers, such as the Oncotype DX DCIS and the 812‐gene classifier, were also predictive in our DCIS‐MIND cohort. This supports the validity of these models, but also shows the possible utility of these classifiers to predict invasive progression.^12^, ^13^

In addition, we developed a three‐dimensional whole‐gland imaging technique to analyse lesion size, location, and growth pattern in three dimensions. Compared to standard two‐dimensional pathology in the clinic, this three‐dimensional approach provides a lot more information, leading to the identification of two distinct DCIS growth patterns, that is replacement or expansive growth. The latter correlated strongly with invasive progression and was more predictive than any other marker. Interestingly, we identified similar three‐dimensional growth patterns in patient specimens. As options to perform three‐dimensional analyses of surgical specimens improve, three‐dimensional pathology of human breast cancers may be a promising avenue to find more indicative biomarkers of DCIS progression.

Sequential transplantation of DCIS‐MIND models revealed remarkable stability over a 3‐year period for features such as growth pattern, molecular subtype, and invasive propensity. Importantly, this effort also yielded a unique collection of 19 distributable DCIS‐MIND models, including OR+/HER2–, OR+/HER2+ and OR–/HER2+ models, together recapitulating the full spectrum of DCIS lesions and vastly expanding the range of models available for DCIS research. Compared to GEMMs, syngeneic allografts, and CDX models, the distributable DCIS‐MIND models better recapitulate the salient features of primary DCIS, such as growth kinetics and molecular subtype. These models are now available to the scientific community for identifying and validating drivers of invasive progression, as exemplified for HER2 overexpression in our study.

**FIGURE 2:**
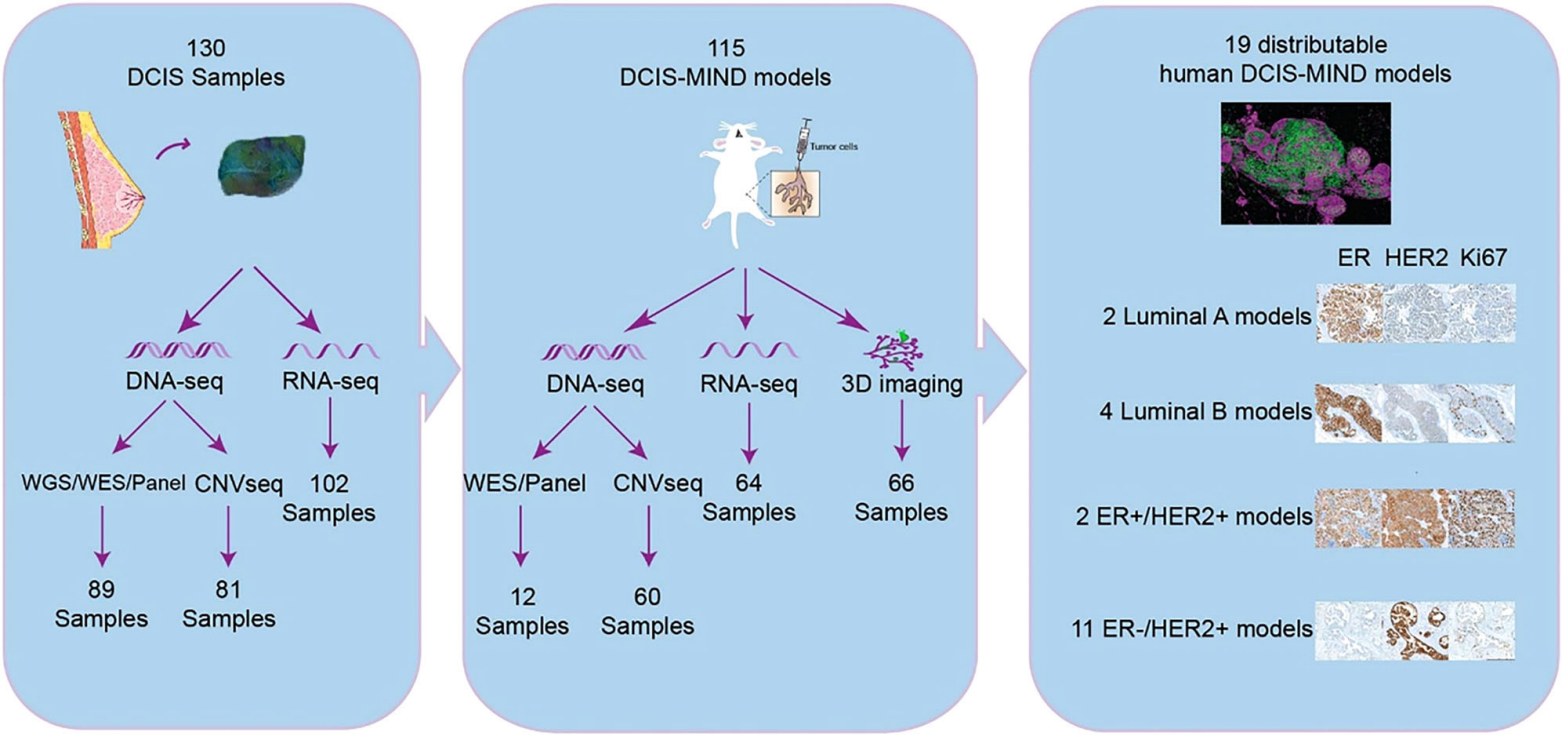
Overview of the multi‐omics and imaging data available for the DCIS‐MIND biobank and the 19 distributable DCIS‐MIND models. DCIS, ductal carcinoma in situ; MIND, Mouse INtraDuctal.

In sum, our study generated a large collection of multi‐omics data of both primary DCIS from patients and DCIS‐MIND models linked to DCIS evolution in mice as well as 19 distributable DCIS‐MIND models (Figure 2). Of note, these DCIS‐MIND models still lack a human immune system and stroma, and could be further improved by incorporating these features using humanised mouse models.^31^, ^32^

## 6 CONCLUSION

**FIGURE 3:**
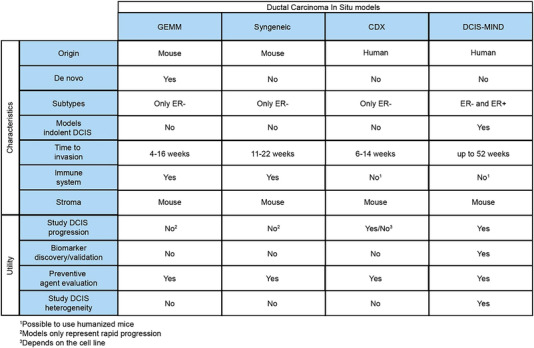
Characteristics comparison between GEMMs, syngeneic, CDX, and DCIS‐MIND models. CDX, cell line‐derived xenograft; DCIS, ductal carcinoma in situ; GEMMs, genetically engineered mouse model; MIND, Mouse INtraDuctal.

Multiple preclinical models of human DCIS have been developed with varying characteristics, as summarised in Figure 3. While all model systems are able to simulate progression of DCIS to invasive disease, most models represent only the most aggressive subtypes of DCIS. These models develop triple‐negative and HER2‐positive DCIS lesions that rapidly progress to invasive disease. Introduction of the MIND method allowed effective modelling of OR‐positive disease and enabled us to create a living biobank of 115 DCIS‐MIND models, which provides a deeper insight in DCIS biology, as well as a collection of 19 distributable DCIS models encompassing both luminal and HER2‐positive subtypes. These 19 models will empower researchers to study the differences between indolent and aggressive DCIS; examine the role of specific genes in DCIS progression; and evaluate the efficacy of novel treatment strategies. Ultimately, the DCIS‐MIND resource provides clinicians and researchers with improved models to further explore the biology of DCIS, which may inform prospective clinical trials designed to prevent overtreatment of DCIS and contribute to more tailored treatments for DCIS patients.

## FUNDING INFORMATION

This work was supported by Cancer Research UK and by KWF Kankerbestrijding (ref. C38317/A24043). Research at the Netherlands Cancer Institute is supported by institutional grants of the Dutch Cancer Society and of the Dutch Ministry of Health, Welfare and Sport.

## CONFLICT OF INTEREST STATEMENT

The authors declare no conflicts of interest.
